# An actinomycete isolate from solitary wasp mud nest having strong antibacterial activity and kills the *Candida* cells due to the shrinkage and the cytosolic loss

**DOI:** 10.3389/fmicb.2014.00446

**Published:** 2014-08-21

**Authors:** Vijay Kumar, Bindu Naik, Omprakash Gusain, Gajraj S. Bisht

**Affiliations:** ^1^Department of Food Technology, Doon P.G. College of Agriculture Science and TechnologyDehradun, India; ^2^Department of Bioprocess and Food Engineering, Institute of Agriculture Sciences, Banaras Hindu UniversityVaranasi, India; ^3^Department of Zoology and Biotechnology, H.N.B. Garhwal UniversitySrinagar, India; ^4^Department of Microbiology, Sardar Bhagwan Singh Post Graduate Institute of Biomedical Sciences and ResearchDehradun, India

**Keywords:** solitary wasp mud nest, *Streptomyces* sp., anti-candidal activity, violaceusniger clade, polyphasic taxonomy

## Abstract

An actinomycetes strain designated as MN 2(6) was isolated from the solitary wasp mud nest. The isolate was identified using polyphasic taxonomy. It produced the extensive branched brown substrate and white aerial hyphae that changed into grayish black. The aerial mycelia produced the spiral spore chains with rugose spore surface. The growth was observed between temperature range of 27–37°C, pH 8–10 and below salt concentration of 6% (w/v). The comparative analysis of 16S rRNA gene sequence and phylogenetic relationship showed that strain MN 2(6) lies in clade with *Streptomyces hygroscopicus* subsp. *hygroscopicus* NRRL 2387^T^, *Streptomyces sporocinereus* NBRC 100766^T^ and *Streptomyces demainii* NRRL B-1478^T^ with which it shares a 16S rRNA gene sequence similarity of 99.3%. The strain MN 2(6) can be differentiated from type strains based on phenotypic characteristics. The strain MN 2(6) showed most promising activity against Gram-positive, Gram-negative bacteria, acid-fast bacilli and *Candida* species suggesting broad-spectrum characteristics of the active metabolite. Evaluation of anti-candidal activity of the metabolite of strain MN 2(6) by scanning electron microscopy (SEM) revealed changed external morphology of yeast. It kills the *Candida* cells due to the shrinkage and the cytosolic loss. However, further studies are required to elucidate the structure of the active metabolite produced by the isolate MN 2(6).

## Introduction

In recent years searching new antibiotics has increased worldwide because of the serious problem of antibiotic resistance among the microbes. The need for new antibiotics has been met largely by the semisynthetic tailoring of natural product scaffolds discovered in the middle of the twentieth century (Clardy et al., 2006). The soil-derived microorganisms have been extensively screened for therapeutically important molecules. However, the frequency of discovering structurally new compounds is apparently decreasing these years and there is a need to seek unutilized microorganisms from unexplored sources (Brady et al., 2002). Moreover, the diversity of secondary metabolites depends more or less on the isolation source, namely, the habitat of the producers (Igarashi, 2004). The recent discovery of the novel primary and secondary metabolites from taxonomically unique population of actinomycetes suggest that these organisms could add a new dimension to microbial natural product research. In this context, new actinomycetes strains producing active compounds have been recently isolated from novel sources including saline, ocean, mangrove forests and niche habitats such as caves, beehives, pristine forests, lakes, rivers, shallow bird, solitary wasp mud nest, and other wetlands (Mukku et al., 2000; Mitra et al., 2008; Promnuan et al., 2009; Radhakrishnan et al., 2010; Poulsen et al., 2011; Kumar et al., 2012a). Assuming the above facts, the actinomycetes of the solitary wasp with regard to the occurrence of novel microbial flora have been studied previously (Kumar et al., 2012a,b). New species of the microorganisms have the potential to produce new metabolites, which justifies the isolation of new species at pharmaceutical research laboratories (Shomura et al., 1979). The present report highlights the taxonomy and antimicrobial activity of a new actinomycete strain isolated from the solitary wasp mud nest, a rare habitat.

## Materials and methods

### Isolation, identification, and characterization

Strain MN 2(6) was isolated from the solitary wasp mud nest collected from Dehradun, India (Kumar et al., 2012a). Cultural characteristics of the strain MN 2(6) was examined every day grown on various International *Streptomyces* project (ISP) media (Shirling and Gottlieb, 1966). Micromorphology and sporulation were observed under light microscope by the inclined coverslip technique (Williams et al., 1989) on ISP-4 medium after incubating at 27°C for 7 days. The spore chain morphology and spore surface ornamentation were examined by scanning electron microscopy (SEM) (Zeiss EVO 40 EP) of 15-day old cultures grown on ISP-4 according to the method described previously (Kumar et al., 2011). Physiological characteristics were examined according to the methods described in the ISP (Shirling and Gottlieb, 1966) and Bergey's Manual of Systematic Bacteriology (Locci, 1989). Resistance to some antibiotics was detected by disc diffusion method. The isomeric forms of diaminopimelic acid (DAP) and the diagnostic sugar in the whole-cell hydrolysates were determined as described in MTCC Laboratory manual, IMTECH (1998), Chandigarh, India.

### Molecular identification

Chromosomal DNA of the strain MN 2(6) was prepared from cells grown in nutrient broth for 2–3 days incubation according to the method described earlier (Kumar et al., 2010). PCR amplification of the 16S rDNA of the strain MN 2(6) was done according to the methods described previously (Kumar et al., 2012a). The identification of phylogenetic neighbors was initially carried out by the BLASTN (Altschul et al., 1997) program against the database containing type strains with validly published prokaryotic names and representatives of uncultured phylotypes (Kim et al., 2012). The top 30 sequences with the highest scores were then selected for the calculation of pair wise sequence similarity using the global alignment algorithm (Myers and Miller, 1988), which was implemented at the EzTaxon-e server (http://eztaxon-e.ezbiocloud.net/; Kim et al., 2012). The isolate was identified using the EzTaxon-e server (http://eztaxon-e.ezbiocloud.net/; Kim et al., 2012) based on 16S rRNA sequence data. The evolutionary history was inferred using the Neighbor-Joining method (Saitou and Nei, 1987). The percentage of replicate trees in which the associated taxa clustered together in the bootstrap test (1000 replicates) are shown next to the branches (Felsenstein, 1985). The tree is drawn to scale, with branch lengths in the same units as those of the evolutionary distances used to infer the phylogenetic tree. The evolutionary distances were computed using the Maximum Composite Likelihood method (Tamura et al., 2004) and are in the units of the number of base substitutions per site. All positions containing gaps and missing data were eliminated from the dataset (Complete deletion option). There were a total of 1376 positions in the final dataset. Phylogenetic analyses were conducted in MEGA4 (Tamura et al., 2007).

### Production, extraction, and partial purification of metabolite

Production of metabolite was done in Glucose soybean meal medium (GS) as described in the previous study (Kumar et al., 2012c). The extraction and partial purification of antimicrobial metabolite from culture filtrate was done according to the method described previously (Kumar et al., 2012c).

### Biological and chemical detection of antimicrobial compounds

Crude extract sample was subjected to thin-layer chromatography (TLC) and spotted onto silica gel plates (Merck Art 5735, Kieselgel 60F254), and then developed with chloroform: methanol: 25% ammonia (1:7:4, v/v) as the solvent mixture. The numbers of antibacterial active fractions were detected by bioautography (Odakura et al., 1984) on silica gel plates seeded with *Micrococcus luteus* and *Candida albicans*. Clear zone of inhibition indicated the position of antimicrobial compounds on the TLC plates, and the retention factor (Rf) value was calculated. The crude product was dissolved in methanol, and the absorption spectrum was recorded at 200–498.8 nm using UV–VIS spectrophotometer (Systronics double beam spectrophotometer). The partial chemical nature of antifungal metabolite was determined using *in vitro* assay. The polyene like activity of isolated antimicrobial metabolite was carried out by ergosterol agar plate method (Jain and Jain, 2006). The antimicrobial compound was partially characterized by spraying with chemical reagents such as 10% KOH ethanolic reagent, Millon's reagent, vanillin-HCl reagent, ninhydrin reagents, iodine vapors, 50% ethanolic H_2_SO_4_, and Dragendorf reagent.

### Determination of antimicrobial activity (MIC method)

Minimum Inhibitory Concentration was determined by the micro dilution method using a 96 well plate according to NCCLS (2008). Micro dilution methods involve the use of plastic microtiter plates (96 well). The two-fold serial dilution of the antibiotics was prepared from 512 to 0.004 μg/ml range (final concentration in Mueller Hinton broth). The MIC is the lowest concentration of the agent which completely inhibits the growth. The control containing no agent should be turbid (negative control) while control with standard antibiotic should be clear (positive control).

### Scanning electron microscopy (SEM) for study of antifungal action of crude extract

The anti-fungal action of extract was carried out by SEM. *C. albicans* was treated *in vitro* with half of the concentrations of MIC crude extract. The cells were harvested after 48 h of incubation at 35°C by centrifugation at 4°C for 5 min and were washed three times with phosphate buffer saline (PBS). The cells pellet was fixed in 3% (v/v) glutaraldehyde in PBS (pH 7.4) and dehydrated in increasing concentrations of ethanol (10%, v/v, increments, to 100%) (Lemar et al., 2005). Cells were mounted onto stubs. The upper surface of each stub was then coated, under vacuum, with a film of gold. The gold coating process was completed in 15–20 min. Once coated with gold, the specimens were ready for examination under scanning electron microscope (ZEISS EVO 40 EP). The gold coated metal stubs were viewed on the SEM at an accelerating voltage of 15 kV, a probe diameter of 102 Pa, to obtain secondary electron images. The field was scanned at low magnification until the line of growth was detected. Areas with clear and cells of yeasts were then selected for examination at higher magnification. Suitable fields in the preparation were photographed.

## Results

**Figure 1:**
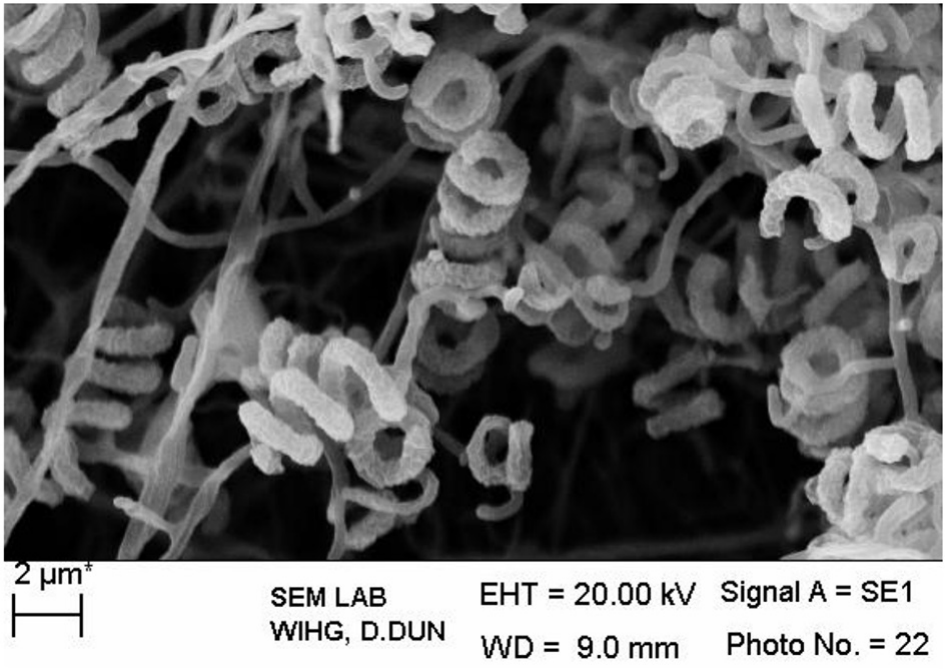
**Scanning electron micrograph showing spore chain morphology of *Streptomyces* sp. MN 2(6); Bar, 2 μm**.

The isolate MN 2(6) was isolated from the solitary wasp mud nest. It produced the extensive branched brown substrate and white aerial hyphae that changed in to grayish black. No diffusible pigments as well as melanin pigments were produced. The SEM revealed that the aerial mycelia produce spiral spore chains. The spore surface was rugose (Figure 1). The cultural characteristics of the *Streptomyces* sp. MN 2(6) are shown in Table 1. It showed growth on all the media except ISP-6 (Table 1). The growth was observed between the temperature range of 27–37°C and pH 8–10 and was found resistant against penicillin G (2 U), co-trimoxazole (25 μg), ciprofloxacin (5 μg), aztreonam (30 μg), cephradine (30 μg), erythromycin (10 μg), and cloxacillin (5 μg). At salt concentration above 6% (w/v), no growth was observed. The physiological characteristics are given in Table 2. Chemotaxonomic tests showed that whole-cell hydrolysates of isolate MN 2(6) were rich in the LL-diaminopimelic acid (LL-DAP), while no characteristic sugar indicated a chemotype I (Williams et al., 1989).

**Table 1:** **Cultural characteristic of *Streptomyces* sp. MN 2(6)**.

**Medium**	**Growth**	**Aerial spore mass**	**Reverse color**	**Diffusible pigment**
Yeast extract-malt extract agar (ISP 2)	Good	White to gray	Brown	^–^
Oat meal agar (ISP 3)	Good	Grayish white	Brown	^–^
Inorganic salt starch agar (ISP 4)	Good	Whitish gray	Green brown	^–^
Glycerol asparagine agar base (ISP 5)	Poor	Gray	Colorless	^–^
Peptone yeast extract iron agar (ISP 6)	Moderate	Whitish gray	Colorless	^–^
Actinomycetes isolation agar (AIA)	Good	White to gray	Brown	^–^
Sabourad dextrose agar (SDA)	Good	Cream	Brown	

–, *Absent*.

**Table 2:** **Phenotypic characteristic of MN 2(6)**.

**Characteristics**	**MN 2(6)**
Aerial mycelium	White to Grayish black
Reverse	Brown
Diffusible pigment	–
Melanin pigment	–
Sporulation	Good
Spore chain	Spirals
Starch hydrolysis	+++
Casein hydrolysis	–
Gelatin hydrolysis	+++
Oxidase	–
Catalase	+
C-utilization	
Dextrose	+++
Rhamnose	–
D-Maltose	++
L-Arabinose	–
L-Sucrose	±
L-Raffinose	+
Cellobiose	++
Fructose	++
Inositol	++
Xylose	++
Salicin	–
D-Mannose	–
Mannitol	++
Trehalose	+
N-utilization	
L-Arginine	–
L-Valine	+++
L-Serine	+++
L-Phenylalanine	+++
L-Threonine	+++
L-Methionine	–
Hydroxyproline	+++
L-Histidine	++
Potassium nitrate	+++
Indole test	–
VP test	–
MR test	–
Nitrate reduction	–
Growth at Mac Conkey	–
H_2_S production	–
Citrate utilization	+++
Degradation of	
Tween 20	–
Tween 40	+++
Tween 80	+++
Tyrosine	+++
Growth at temp.	
4–10°C	–
15°C	–
20–37°C	++
Growth at NaCl (w/v)	
0–6%	+
Growth at pH	
4	+
5	+
9	+
10	+
12	+
Growth in presence	
Crystal violet (0.001, w/v)	++
Phenol (0.1%, w/v)	–
Pottasium terrulite (0.001%, w/v)	++
(0.01%, w/v)	++
Sodium azide (0.01%, w/v)	++
(0.02%, w/v)	++

+++, excellent growth;

++, moderate growth;

+, poor growth;

–, no growth;

±, *doubtful*.

**Figure 2:**
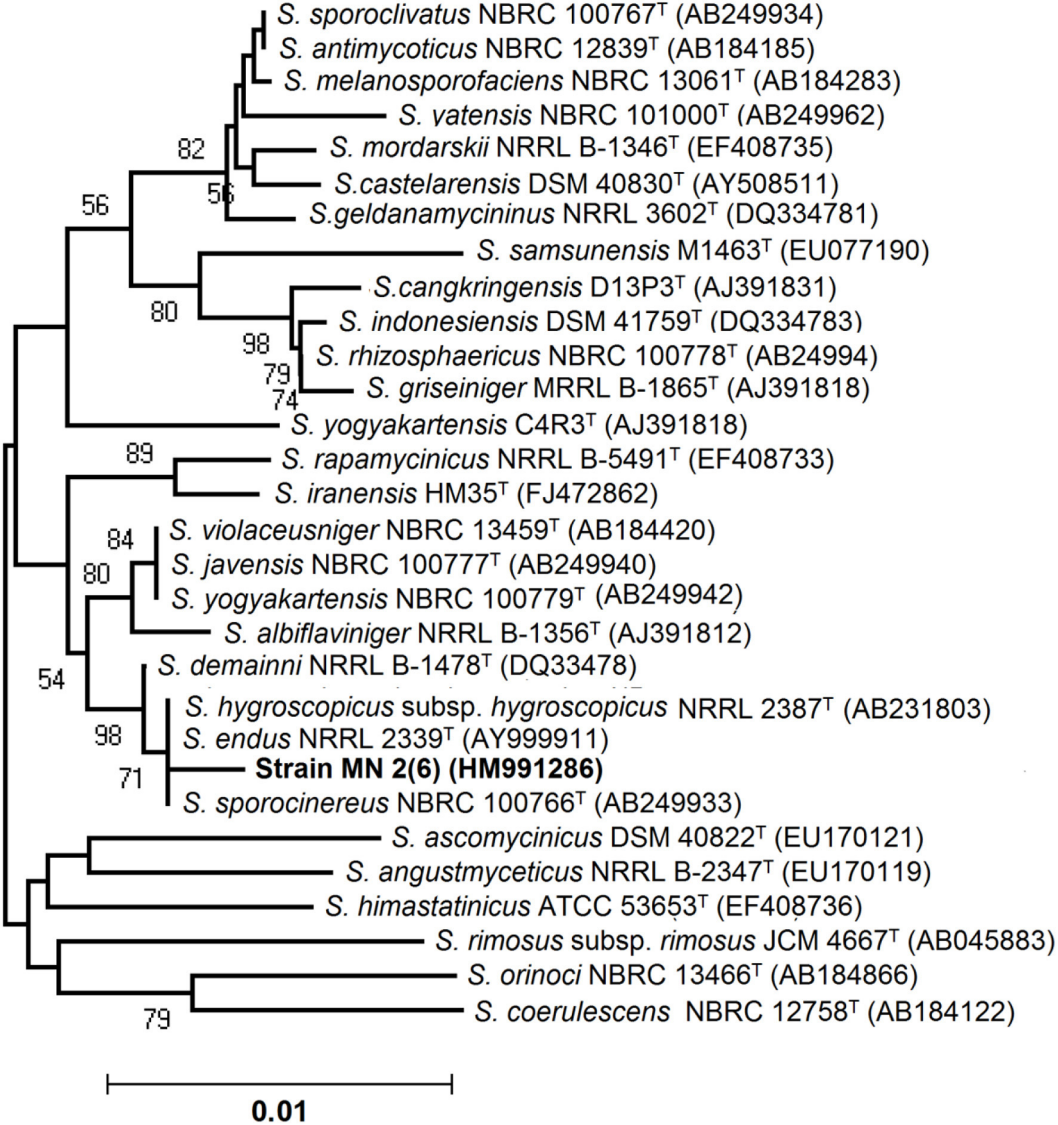
**Neighbor-joining tree showing the relationship between strain MN 2(6) and closely related members of the genus *Streptomyces* based on 16S rRNA gene sequences**. Bootstrap percentages based on 1000 resamplings are listed at Nodes; only values above 70% are given. Bar 0.01 changes per nucleotide position. T indicates the type strains.

An almost complete 16S rRNA gene sequence (1419 nt) for isolate MN 2(6) was determined in this study and submitted in Genbank under the accession number HM991286. The sequence was first analyzed by BLAST search and was then aligned with those of *Streptomyces* reference strains available in the Genbank database, which confirmed its identification as isolate MN 2(6) at genus level. Its position among the type strains of *Streptomyces* is shown in Figure 2. A phylogenetic tree was constructed based on 16S rRNA gene sequences to show the comparative relationship between strain MN 2(6) and other related *Streptomyces* species (Figure 2). The comparative analysis of 16S rRNA gene sequence and phylogenetic relationship showed that strain MN 2(6) lies in clade with *S. hygroscopicus* subsp. *hygroscopicus* NRRL 2387^T^, *S. sporocinereus* NBRC 100766^T^, and *S. demainii* NRRL B-1478^T^ with which it shares a 16S rRNA gene sequence similarity of 99.3%.

**Figure 3:**
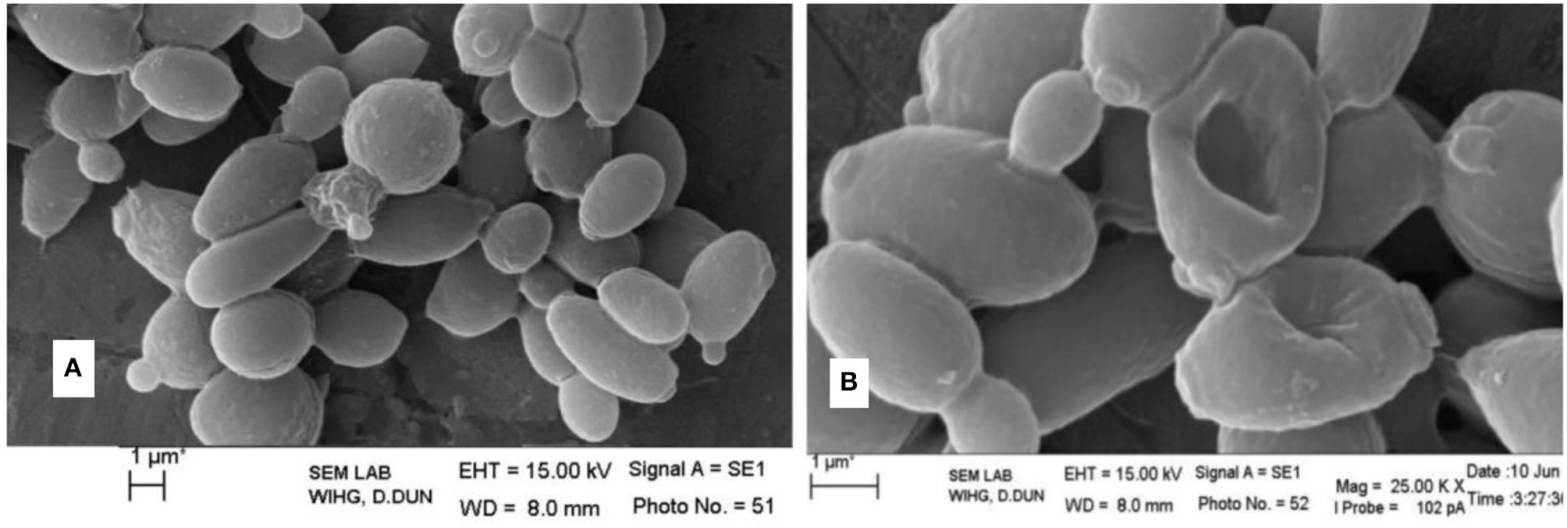
**Effect of antifungal metabolite on *Candida albicans* cells; (A) without treatment; (B) treatment with active metabolite produced by MN 2(6)**.

The antimicrobial activity of the strain MN 2(6) is given Table 3. The strain MN 2(6) inhibited Gram-positive bacteria, Gram-negative bacteria and yeast, suggesting broad-spectrum characteristics of the active metabolite. Lowest MIC value was recorded against *M. smegmatis* MTCC 6 (0.25 μg/mL), *S. aureus* MTCC 2940 (1.0 μg/mL), and *Acinetobacter baumanii* MTCC 1425 (1.0 μg/mL) followed by *S. aureus* MTCC 96 (2.0 μg/mL), *S. aureus* clinical-1 (β-lactamase) (2.0 μg/mL), *E. faecalis* MTCC 439 (2.0 μg/mL), *Candida parapsilosis* (2.0 μg/mL), *C. albicans* MTCC 1637 (2.0 μg/mL), and *Candida tropicalis* MTCC 2208 (2.0 μg/mL) while other pathogens showed higher value of MIC (Table 3). The partially purified product extracted with resin (Dianion™ HP-20) was checked for the number of bio-active compounds present in the methanol extract. One bio-active compound was found to have a Rf value of 0.78 (Figure S1). The chromogenic reactions were negative with 10% KOH ethanolic reagent, Millon's reagent, Dragendof reagent, and vanillin-HCl reagent, suggesting the absence of anthraquinones, phenol glycosides, heterocyclic compounds and myrrh constituents. However, it showed positive reactions with iodine vapors, 50% ethanolic H_2_SO_4_, and ninhydrin, indicating the presence of conjugated double bond, cardiac glycoside and free amine groups. The characteristics peak at 209 nm (Figure S2) indicates no chances of polyene class of antibiotics, which was further confirmed by ergosterol test. Evaluation of anti-candidal activity of the metabolite of strain MN 2(6) by SEM revealed control cells of *C. albicans* after 48 h of incubation showing normal oval shapes with smooth surfaces (Figure 3A). Control cells were lying apart, showing polar buds and bud scars, treatment of *C. albicans* at concentration of ½ MIC crude extract changed the external morphology of yeast (Figure 3B). The external morphology of the cells did not appear as smooth as that of untreated cells.

**Table 3:** ***In vitro* MICs (μg/ml) of MN 2(6), Van, Rif, AmB against various bacterial pathogens and fungal pathogens by broth dilution method**.

**Organism**	**MN 2(6)**	**Van**	**Rif**	**AmB**
*S. aureus* MTCC 2940	1	2	4	NA
*S. aureus* MTCC 96	2	0.5	0.5	NA
*S. aureus* clinical-1 (β-lactamase)	2	1	0.5	NA
*S. aureus* clinical-2 (β-lactamase)	5	1	0.25	NA
*S. aureus* clinical-3 (β-lactamase)	5	1	0.12	NA
*B. subtilis* MTCC 441	5	4	2	NA
*M. luteus* MTCC 106	5	4	0.01	NA
*E. coli* MTCC 739	25	–	64	NA
*E. coli* MTCC 2939	5	–	1	NA
*E. coli* clinical	50	–	16	NA
*P. aeruginosa* MTCC 424	512	–	64	NA
*P. aeruginosa* clinical-1	512	–	128	NA
*P. aeruginosa* clinical-2	512	–	64	NA
*Salmonella* sp.	512	512	32	NA
*Acinetobacter junii* MTCC 1686	0.5	1	0.03	NA
*Acinetobacter baumanii* MTCC 1425	1	256	8	NA
*E. faecalis* MTCC 439	2	1	4	NA
*M. smegmatis* MTCC 6	0.25	16	8	NA
*Candida parapsilosis*	2			2
*Candida albicans* MTCC 1637	2			1
*Candida tropicalis* MTCC 2208	2			2.5

*Product of Streptomyces sp. MN 2(6); Van, vancomycin sulfate; Rif, rifampicin; AmB, amphotericin-B, –, not active; NA, not applicable*.

## Discussion

Microorganisms have served as a source for the majority of the drugs in use today (Demain and Snchez, 2009). Among the different microbes, actinomycetes have been and are the unparalleled source of bioactive metabolites. The metabolites from natural sources continue to play a key role in drug discovery and development by providing natural molecules with pharmaceutical potential and novel scaffolds for synthetic modification (Vincet et al., 2006). In this context, the isolate MN2 (6) of the previous study was taken for further studies. Based on cultural, physiochemical and chemo-taxonomical characteristics, it was found to belong the genus *Streptomyces*. The comparative analysis of 16S rRNA gene sequence and phylogenetic relationship showed that strain MN 2(6) lies in clade with *S. hygroscopicus* subsp. *hygroscopicus* NRRL 2387^T^, *S. sporocinereus* NBRC 100766^T^ and *S. demainii* NRRL B-1478^T^ with which it shares a 16S rRNA gene sequence similarity of 99.3%. The members of violaceusniger clade shared 16S rRNA gene similarities within the range 99.0–99.8%, values that correspond to between 3 and 11 nt differences at 1449 and 1446 locations, respectively. The highest 16S rRNA similarity found between the type strains belonging to this taxon a value equivalent to 7 nt differences at 1447 sites. Extensive DNA–DNA relatedness experiments indicated that members of the *S. violaceusniger* 16S rRNA gene clade that share similarities at or below 99.7%, that is, with four or more nucleotide differences, can be assigned to different genomic species (Goodfellow et al., 2007). As MN 2(6) shared 99.37% sequence similarity with type strains with nine nucleotide differences, hence may be novel species of *Streptomyces* belonging to violaceusniger clade. Although, strain MN 2(6) shares high 16S rDNA similarity value with *S. demainii* NRRL B-1478^T^, *S. endus* NRRL 2339^T^, *S. sporocinereus* NBRC 100766^T^, and *S. hygroscopicus* subsp. *hugroscopicus* NRRL 2387^T^ but differs in various phenotypic characteristics. MN 2(6) produced white to whitish gray aerial and brown reverse mycelium while *S. sporocinereus* (Gause et al., 1983) produced sparse white aerial mycelium and green beige reverse mycelium. The spore surface of *S. sporocinereus* was smooth while it was rugose in case of MN 2(6). *S. sporocinereus* tolerate a salt concentration of 2.5% (w/v) whereas MN 2(6) can tolerate a salt concentration up to 6% (w/v). Xylose, inositol, raffinose and cellobiose are not utilized by of *S. sporocinereus* while the same was utilized by MN 2(6). Arginine was utilized by the type strains where as MN 2(6) does not utilize it. These characteristics differentiate MN 2(6) with type strain of *S. sporocinereus*. Similarly, MN 2(6) was differentiated from *S. demainii* (Goodfellow et al., 2007) in a number of characteristics. *S. demainii* produced gray aerial mycelium and grayish yellow reverse mycelium, reduced nitrate and hydrolysed casein whereas MN 2(6) did not give positive results for nitrate reduction and casein hydrolysis. Arabinose and salicin were utilized by *S. demainii* whereas as MN 2(6) did not utilize the same. Fructose and L-Histidine were not utilized by *S. demainii* while the same was utilize by MN 2(6). *S. demainii* grew at pH 4, 5, 9, and 10 only while MN 2(6) grew at a wide range of pH (4–10). MN 2(6) was also differentiated from *S. hygroscopicus* subsp. *hugroscopicus* (Goodfellow et al., 2007) in cultural characteristics, nitrate reduction and casein hydrolysis properties. The type strain of *S. hygroscopicus* subsp. *hugroscopicus* grew only at pH 5 whereas MN 2(6) grew at a pH range of 4–10. From the above discussion, clearly MN 2(6) is different from the type strains and may be novel species of *Streptomyces* belonging to violaceusniger clade.

The result of antimicrobial activity is comparable with the previous study (DeBoer et al., 1970; Furumai et al., 2003) and *Streptomyces* sp. MN2 (6) showed promising activity against Gram-positive, Gram-negative bacteria and *Candida* species. The result of bioautography indicates the presence of only one bioactive compound. The chemical characterization of this active compound suggests the absence of anthraquinones, phenol glycosides, heterocyclic compounds and myrrh constituents. However, it indicates the presence of conjugated double bond, cardiac glycoside and free amine groups (Kumar et al., 2012c). The characteristics peak at 209 nm indicates no chances of polyene class of antibiotics (Jain and Jain, 2006). However, the metabolite contained carboxy or peptide moiety in the compound as it showed maximum absorbance between 205 and 216 (Singh et al., 2009). Evaluation of anti-candidal activity of the metabolite of strain MN 2(6) by SEM may be helpful to understand the cell damage mechanism. The changes in morphology of yeast cells after treatment with *Streptomyces* sp. MN 2(6) are also consistent with findings of other researchers (Kitajima et al., 1976; Nurkanto and Julistiono, 2014). The shrinkage of cells was clearly observed in the electron micrograph which may be due to loss of cytosolic volume, which is mainly observed in case of polyene class of antibiotics (Kitajima et al., 1976). However, preliminary characterization of metabolites indicates that it is a non-polyene class of metabolite. These findings support that the stains MN 2(6) possess strong anti-candidal activity and killed pathogenic yeast due to considerable morphological changes.

From the results of the present study, it was concluded that the isolate may represent a novel species of *Streptomyces* belonging to violaceusniger clade and produce metabolite (s) which has a broad spectrum (active against Gram-positive, Gram-negative bacteria, acid-fast bacteria and yeast cells). It kills the *Candida* cells due to the shrinkage and cytosolic loss. However, further studies are required to elucidate the structure of the active metabolite produced by the isolate MN 2(6).

### Conflict of interest statement

The authors declare that the research was conducted in the absence of any commercial or financial relationships that could be construed as a potential conflict of interest.
