# Supplementary material for: An actinomycete isolate from solitary wasp mud nest having strong antibacterial activity and kills the Candida cells due to the shrinkage and the cytosolic loss
Source: Front Microbiol. 2014 Aug 21;5:446. doi: 10.3389/fmicb.2014.00446 (PMC4140075; doi:10.3389/fmicb.2014.00446)
Supplement: Supplementary file 1 [file DataSheet1.DOCX]

**Supplementary Material**


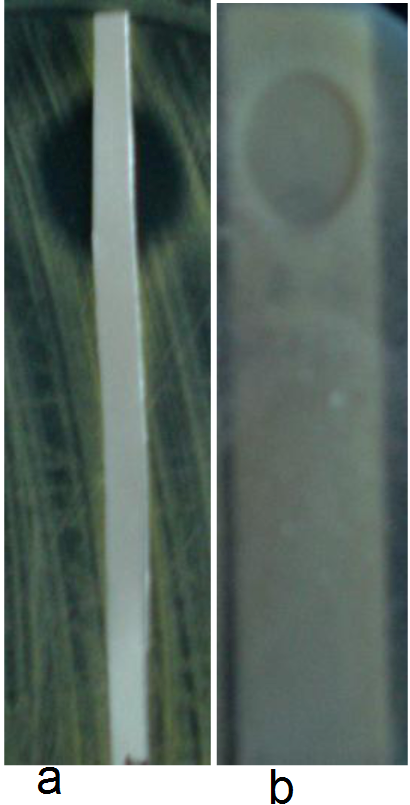


**Fig. S1** Detection of active principle using TLC, bioautogram of crude product isolated from strain MN 2(6) developed with: a, *Micrococcus* *luteus*; b, *Candida* *albicans*


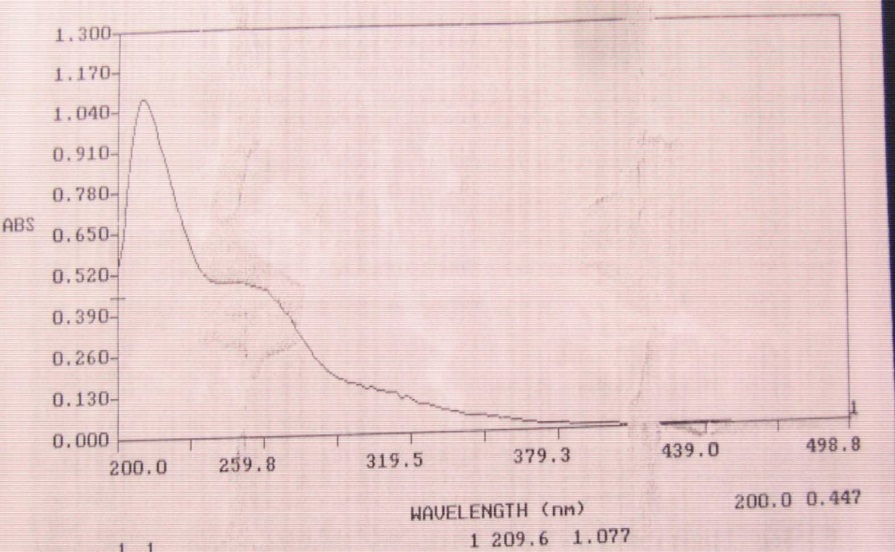


**Fig .S2** UV–VIS spectra of resin extracted metabolite produced by *Streptomyces* sp. MN 2(6)
